# Cutaneous Mixed Tumour: A Rare Presentation of a Scrotal Lump Mimicking an Accessory Testis

**DOI:** 10.7759/cureus.49590

**Published:** 2023-11-28

**Authors:** Feras T Tomalieh, Teresa Rovira, Katie McComb, Raveendra Surange, Patrick Shenjere, Nyla Nasir

**Affiliations:** 1 Urology, Royal Oldham Hospital, Oldham, GBR; 2 Histopathology, Royal Oldham Hospital, Oldham, GBR; 3 General Surgery, North Manchester General Hospital, Manchester, GBR

**Keywords:** skin tumours, cutaneous mixed tumour, syringoma, accessory testis, chondroid syringoma

## Abstract

Cutaneous mixed tumour (CMT), also known as chondroid syringoma (CS), is a rare benign tumour composed of epithelial, myoepithelial, and mesenchymal components with an incidence of less than 0.01% of primary skin tumours. It is more common in males and typically presents as a painless slow-growing firm mass in the subcutis of the head and neck region. Genital regions are very rarely involved. We present the case of a 50-year-old male with a 10-year history of an asymptomatic gradually enlarging mass in the upper scrotum. A surgical excision was performed. Microscopic examination showed features of CMT. This case highlights the diagnostic challenges associated with scrotal CMT and surgical management of these lesions. Additionally, we endorse the recommended terminology of CMT used by the fifth edition of WHO Classification of Skin Tumours (2023).

## Introduction

Cutaneous mixed tumour (CMT) is a rare benign mixed tumour of the skin composed of epithelial, myoepithelial and mesenchymal components with an incidence of less than 0.01% of all primary skin tumours [1]. CMTs most commonly occur in the head and neck region [1-3]. Genital regions are very rarely involved. We present a rare case of CMT presenting as a scrotal mass.

## Case presentation

**Figure 1:**
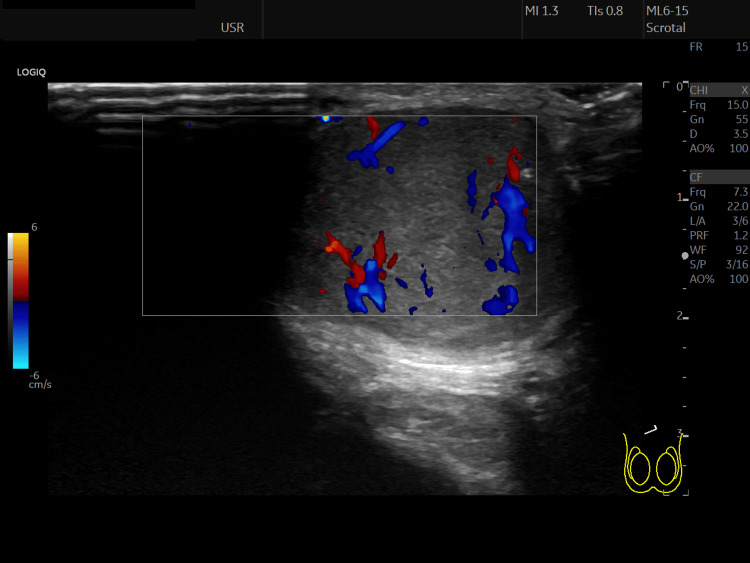
Ultrasound of soft tissue scrotal lesion

**Figure 2:**
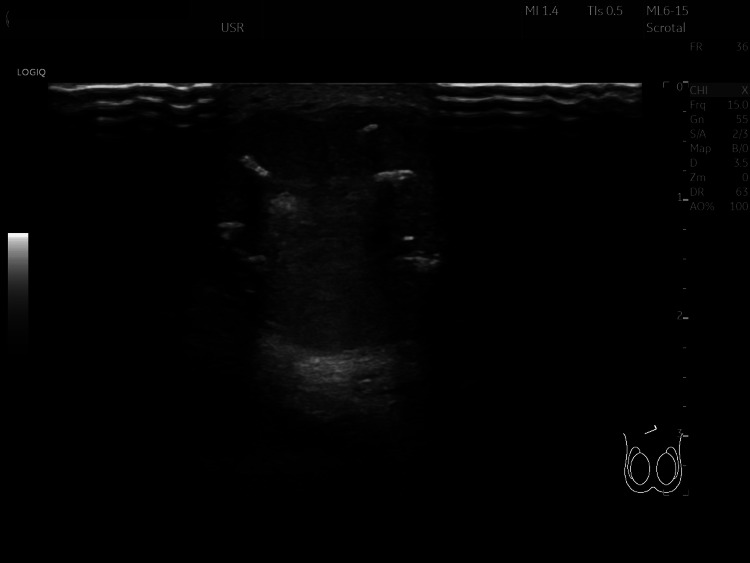
Ultrasound of soft tissue scrotal lesion

A 50-year-old Caucasian male presented to the Urology outpatient clinic with a 10-year history of a scrotal mass that had gradually increased in size. There was no significant past medical history. Clinical examination revealed a well-circumscribed 50mm solid lump in the upper scrotum at the root of the penis. This was felt to be subcutaneous and not related to the testicle or spermatic cord. Tumour markers were not performed as the mass was not related to the testis. On ultrasound the testis and epididymis were normal bilaterally in size shape echotexture and vascularity with no focal lesions. The soft tissue scrotal mass was considered to be an accessory testis (Figures 1, 2).

**Figure 3:**
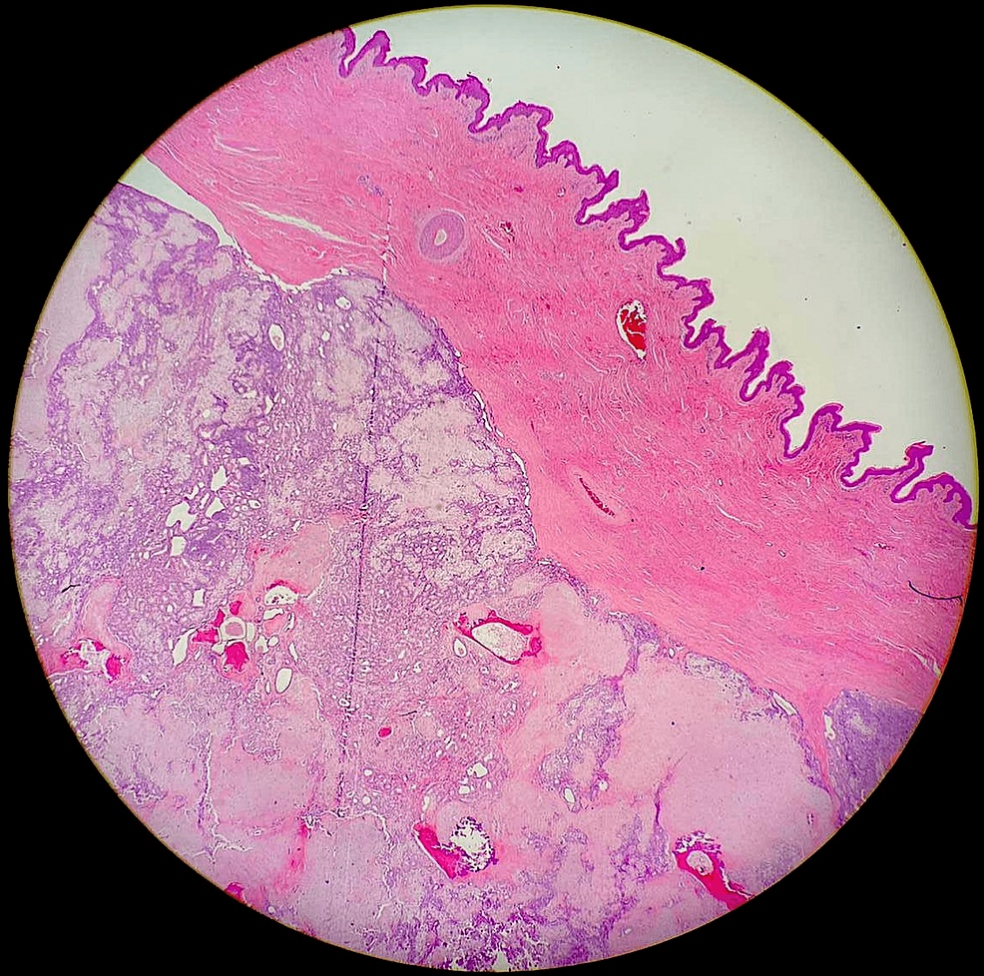
High-resolution histology image demonstrating scrotal skin (on the right) with a well-circumscribed, partly encapsulated lobulated lesion (on the left)

**Figure 4:**
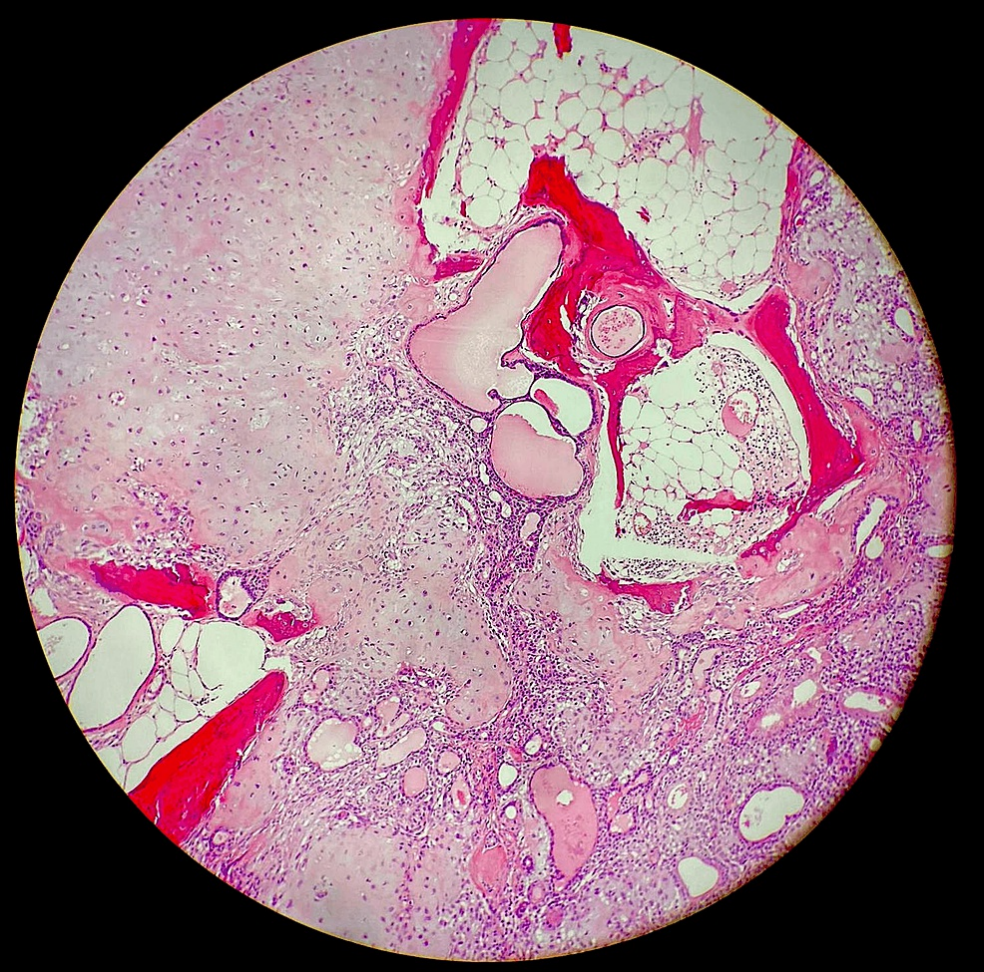
Epithelial cells forming ducts and tubules with surrounding lacelike network of myoepithelial cells in a chondromyxoid stroma with areas of osseous and adipose tissue components

**Figure 5:**
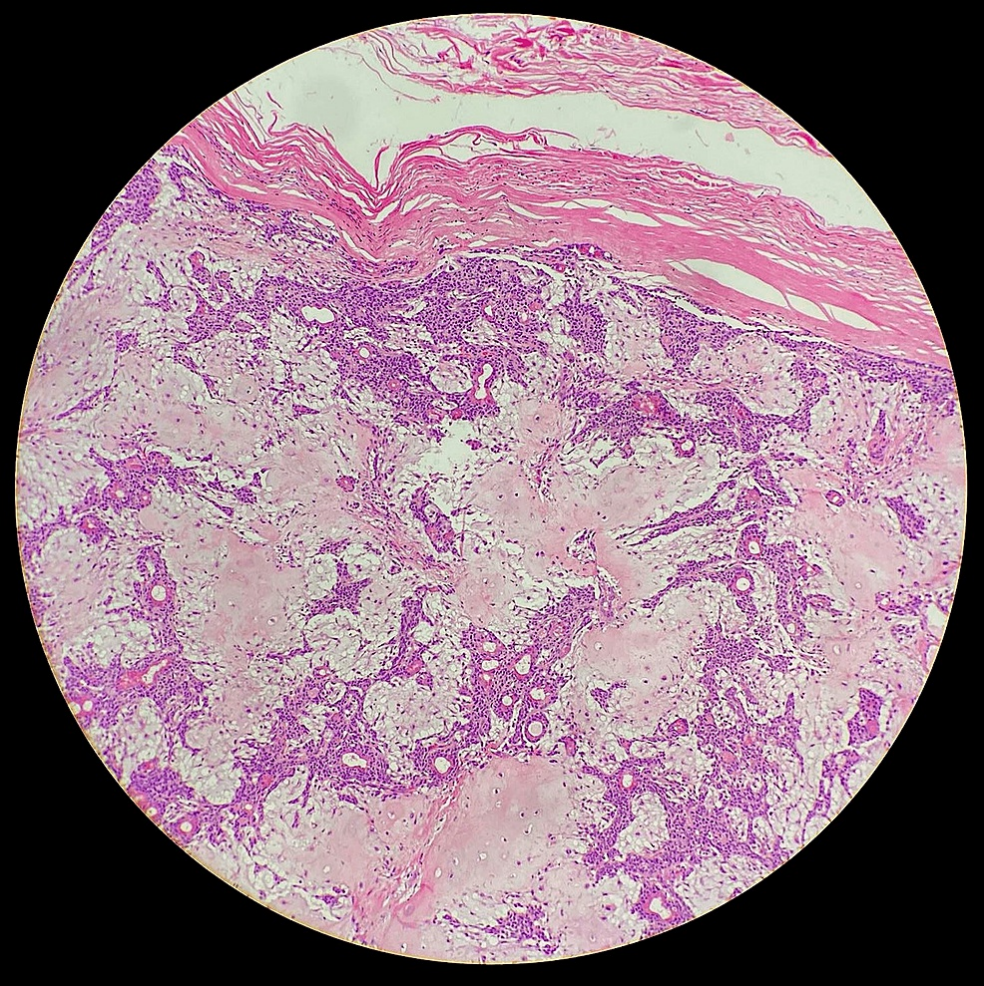
Branching ductal structures composed of bland-appearing epithelial cells with surrounding myoepithelial cells

**Figure 6:**
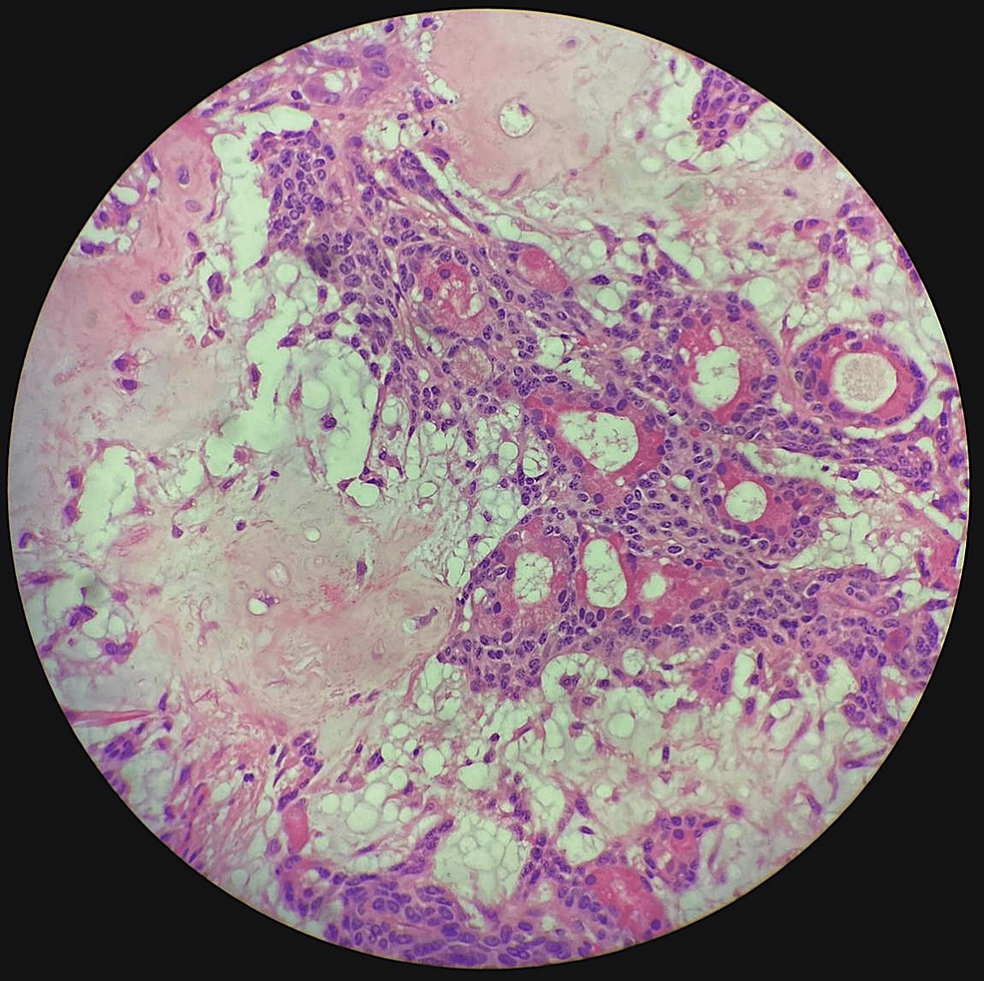
HP view of ducts surrounded by myoepithelial cells

The mass was excised and sent for histopathology. Macroscopic examination showed a well-circumscribed 52x35x32mm predominantly subcutaneous soft nodule with overlying skin. Microscopic assessment showed a well-circumscribed partly encapsulated multilobulated lesion located within the deep dermis and subcutaneous fat. The tumour had a biphasic appearance comprising a dual population of bland epithelial cells forming ducts and tubules with surrounding myoepithelial cells forming a reticular lace-like network. The stroma was partly myxoid with areas of chondroid, focal osseous differentiation and mature adipose tissue formation (Figures 3-6).

**Figure 7:**
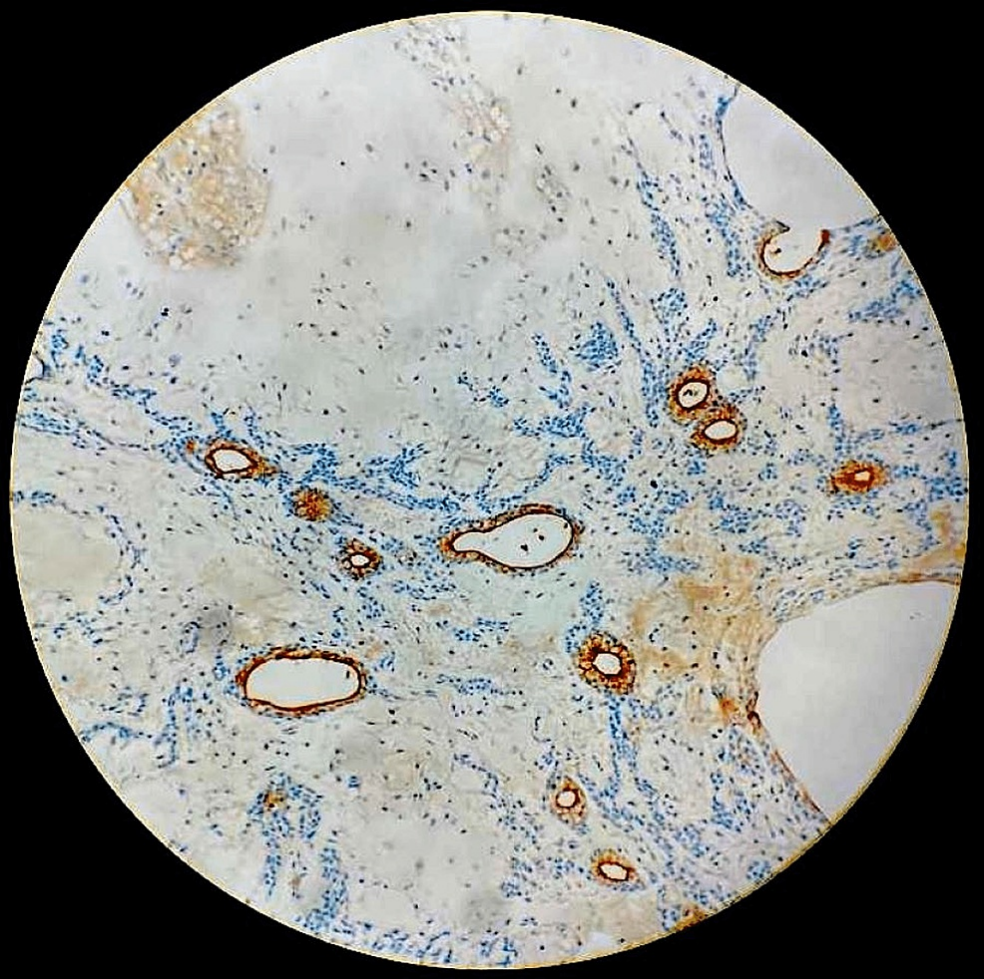
Carcinoembryonic antigen (CEA) immunostain highlighting tubules and ducts

**Figure 8:**
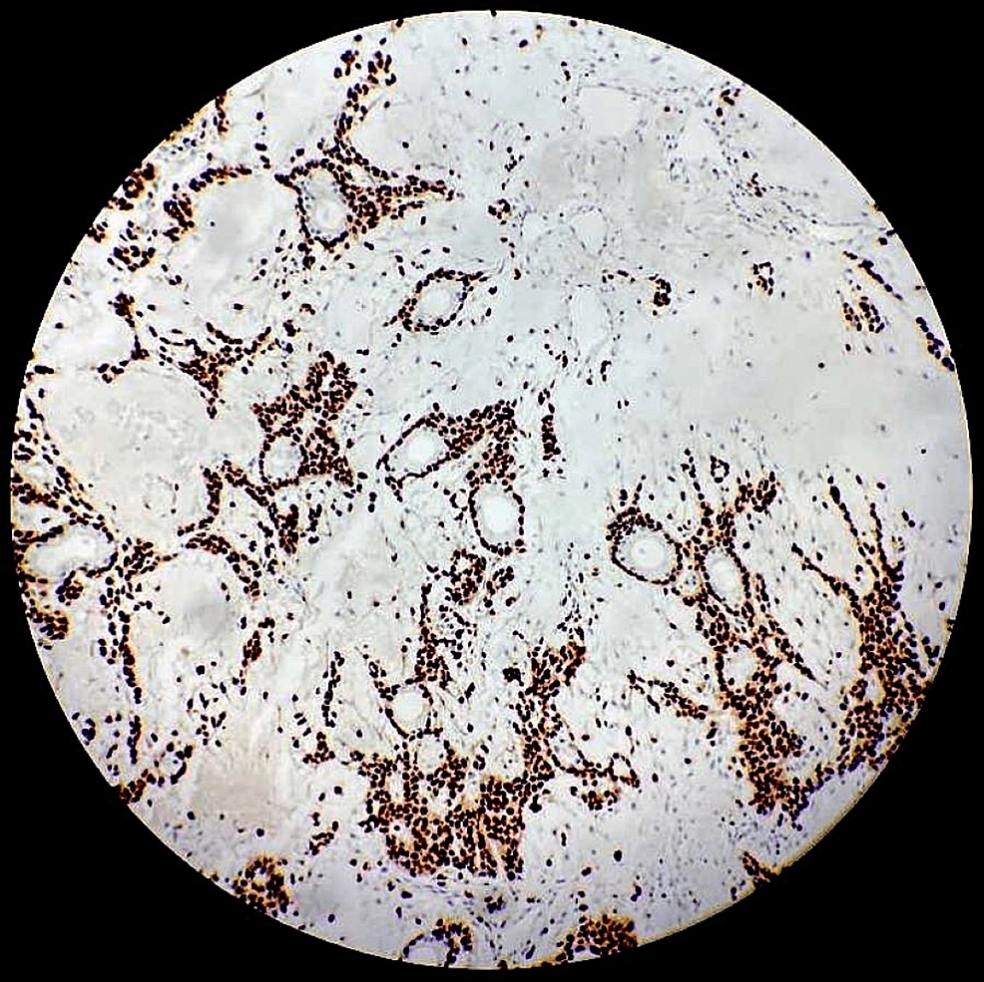
SRY-related HMG-box 10 (SOX10) immunostain marks myoepithelial cells surrounding the tubules

On immunohistochemistry (IHC) the ductal epithelial cell population was positive for epithelial markers MNF-116, carcinoembryonic antigen (CEA) and EMA. The myoepithelial cell population was positive for SRY-related HMG-box 10 (SOX10), SMA, S100 and CK14 (Figures 7, 8).

There was no evidence of infiltration into surrounding tissue or satellite nodule formation, and no cellular pleomorphism or necrosis was seen, to suggest malignancy. The case was reported as benign tumour consistent with CMT. Owing to the rarity of a CMT in this location the case was sent to a soft tissue expert who concurred with the diagnosis.

The lesion did not involve the surgical resection margins confirming that a clear excision was achieved. However, owing to nearby anatomical structures only a narrow margin of uninvolved tissue could be included in the resection. Ongoing outpatient follow-up will be undertaken to monitor for recurrence with clinical review, examination and imaging if required.

## Discussion

CMTs are benign tumours of the skin located within the dermis or subcutaneous tissue and were previously known as chondroid syringoma (CS). These tumours are morphologically similar to the benign mixed tumour (pleomorphic adenoma) of salivary glands [4-6].

The term CS was first suggested by Hirsch and Helwig in 1961 for mixed tumours of the skin following a case series study of tumours with benign epithelial, myoepithelial and mesenchymal elements in a chondromyxoid matrix, considered to be derived from sweat glands of the skin [7]. CMT is the recommended terminology for these tumours used by the fifth edition of the WHO Classification of Skin Tumours (2023).

CMTs present as a slow-growing solitary painless nodule without ulceration [4]. On palpation they usually feel firm and well-circumscribed [1,8]. Typically these tumours arise in the head and neck region [2-4]. Rare cases have been reported presenting in the skin of abdomen, axilla, penis, vulva, scrotum, hands and feet [5,7,9]. It is more common in men than women [3]. The age range is from 20 to 60 years [9].

To the best of our knowledge, only 10 previous cases of scrotal CMT have been reported [10,11]. Hence, there is limited awareness and experience of the presentation of these lesions in this location. The tumour is generally asymptomatic and grows gradually, hence the patient tends to present to clinicians relatively late usually due to cosmetic reasons or trauma [7,9,10]. In a study by Okuda et al., the average time described in the literature from initial presentation to elective excision is 7.9 years with average size of tumour at time of excision of 42mm [10]. In our case the clinical course was 10 years prior to excision, and the lesion measured 52mm.

A clinical diagnosis of scrotal CMT is challenging due to its rarity and clinical similarity to other benign scrotal masses [9]. Differential diagnoses include lipomas, dermatofibromas, neurofibromas, pilar cysts and epidermal inclusion cysts [9]. In our case, the lesion was thought to be an accessory testis on review of ultrasound imaging.

The diagnosis of CMT is usually not made on preoperative clinical or radiological findings. Histopathological analysis of the excised lesion remains the mainstay for the diagnosis of CMT [2,4,9-11]. CMTs are mixed tumours with multiple components, and depending on the area sampled not all components may be represented in the excised material [2,9]. Sampling limitations may lead to a misdiagnosis.

The characteristic histological features include epithelial elements comprising of cuboidal or polygonal cells forming nests, ductal structures and intercommunicating tubulo-alveolar structures admixed with myoepithelial cells in a chondromyxoid matrix and occasional keratinous cysts. Mesenchymal elements if present show osseous, chondroid and adipocytic differentiation [2,3,5,7]. On histochemical stains the chondromyxoid areas are positive for mucicarmine, periodic acid-Schiff (PAS) and Alcian Blue (AB), indicating it be acid mucopolysaccharides found in normal cartilage [4,7].

Two histopathologic subtypes have been described. CMT of apocrine origin is the more common subtype, which exhibits ducts with lumina lined by two layers of epithelial cells, decapitation sections similar to apocrine glands are present [4,12]. The architecture is complex with branching and anastomosing ductal structures. Those of eccrine origin, which are less common, have a simpler architecture with tubules lined by a single layer of flattened cuboidal epithelial cells [1,4,6]. Similar to pleomorphic adenomas, PLAG1 rearrangement and overexpression by IHC have been previously demonstrated in a subset of CMTs [6]. Recent evidence shows that eccrine and apocrine CMTs have a different molecular pathogenesis, in which PLAG1 gene overexpression was observed in the majority of apocrine but not eccrine CMTs [6]. To date no prognostic differences between the two subtypes have been reported.

IHC can be used as an adjunct to support the diagnosis of CMT. The ductal and tuboalveolar elements are positive for epithelial markers pancytokeratin, CEA and EMA [10,11,13]. The stromal cells are positive for myoepithelial markers SOX10, CK14 and p63 [10,11,13]. Stromal cells are also positive for SMA, vimentin, S-100 and GFAP [10,11,13].

Although CMTs are predominantly benign, malignant forms have been reported. Malignant CMTs may originate de novo, in recurrent lesions or rarely arise in an existing benign mixed tumour which may suddenly undergo malignant change [7,13,14]. Features of malignancy are best assessed in an excision specimen. Malignant tumours tend to be larger than 30mm; they show cellular pleomorphism, nuclear atypia, mitoses and necrosis with evidence of local invasion in the form of infiltration or satellite nodules [14].

Treatment of CMT involves complete surgical excision with an appropriate margin of normal tissue [2,8]. This ensures the entire lesion is removed which is essential to reduce the risk of recurrence [1,5,8,10,11]. Recurrence of these tumours most likely results from either inadequate excision or satellite tumour islands [7,13,14]. Malignant changes may develop in the recurrent tumours [13]. Hence clinical follow-up and monitoring are essential following surgical excision of CMT. If malignant recurrence occurs, the mainstay of treatment is wide surgical excision [15]. The utilisation of radiotherapy has been reported in the presence of nodal metastases [15].

## Conclusions

Scrotal CMT is a rare benign tumour. It should be considered within the differential diagnosis of a slow-growing well-circumscribed mass within the subcutaneous scrotal skin. Radiological imaging can assess the extent and depth of the lesion as well as relation to adjacent anatomic structures however there are no specific features to indicate CMT. Definitive diagnosis and exclusion of malignancy is only possible on histopathological assessment. Owing to the lobulated contours of the tumour, surgical excision with margins of normal surrounding tissue is essential. This reduces the risk of local recurrence and is considered to be an effective treatment.
